# Analysis of the Thermal Oxidation of Biodiesel with and without Coffee Leaf Extract Using Nuclear Magnetic Resonance (^1^H NMR)

**DOI:** 10.1021/acsomega.5c02859

**Published:** 2025-08-16

**Authors:** Ana Carolina G. Mantovani, Érica S. Romagnoli, Jose Gonçales Filho, Renan A. Sartor, Isadora G. Branco, Lilian F. S. Tupan, Daniel F. Valezi, Anuar J. Mincache, Dionísio Borsato, Karina B. Angilelli

**Affiliations:** a Engineering Department, Ingá University Center (Uningá), Rod. PR 317, 6114 Parque Industrial 200, Maringá, PR 87035-510, Brazil; b Chemistry Department − LPAC, 37894 State University of Londrina (UEL), Rd. Celso Garcia Cid, Pr 445, Km 380, Londrina, PR 86057-970, Brazil; c Department of Physics and Mathematics, São Paulo State University (UNESP), Institute of Chemistry, Prof. Francisco Degni Street, 55, Araraquara, SP 14800-060, Brazil

## Abstract

Biodiesel is a renewable and biodegradable alternative fuel, but its susceptibility to oxidative degradation compromises its storage stability and performance. Synthetic antioxidants are commonly used to mitigate this issue. However, there is growing interest in natural antioxidants as sustainable alternatives. This study aimed to investigate the thermal-oxidative degradation of pure biodiesel (B100) and biodiesel with coffee leaf extract (B100E) using ^1^H NMR spectroscopy as well as to correlate the iodine value (IV) with NMR spectra and monitor the formation of oxidation products throughout the degradation process. Biodiesel samples underwent accelerated oxidation at 110 °C using the Rancimat method, followed by ^1^H NMR analysis to identify oxidation products. The degradation kinetics indicated that linolenate and linoleate compounds were oxidized faster than oleate groups, leading to an increase in the number of saturated compounds. A linear correlation was found between the percentage of olefinic hydrogen atoms from the ^1^H NMR spectra and the iodine value determined by the Wijs method. The addition of coffee leaf extract effectively delayed oxidation, as evidenced by the slower appearance of oxidation products and a reduced increase in saturated compounds. These findings highlight a novel and efficient methodology for evaluating biodiesel degradation and unsaturation by combining ^1^H NMR analysis with iodine value determination. In addition to enabling a rapid, nondestructive estimation of IV, ^1^H NMR also allowed the characterization of different stages of oxidative degradation through the identification and monitoring of specific oxidation products over time.

## Graphical abstract

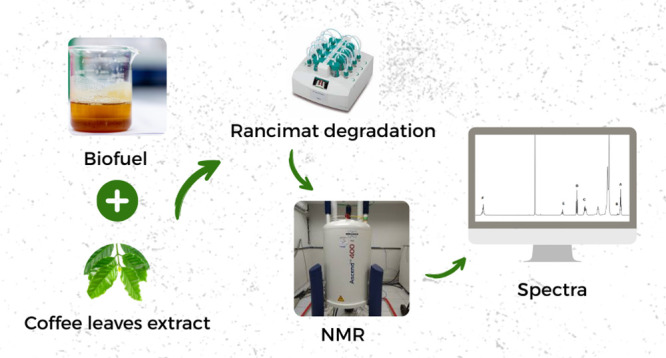

## 1 Introduction

Biodiesel is a renewable and biodegradable alternative fuel to diesel. It offers several environmental advantages; however, its susceptibility to oxidative degradation presents a significant challenge in its storage and use.^1^

When exposed to factors such as heat, radiation, and air, biodiesel undergoes a complex oxidation process, leading to the formation of products such as hydroperoxides, epoxies, aldehydes, and ketones.^2–4^ These oxidation products negatively affect the biofuel’s physicochemical properties, increasing its viscosity and acidity, while accelerating the formation of gums, which can damage engines.^5^

The oxidation of biodiesel follows an irreversible radical reaction divided into three main stages: initiation, propagation, and termination.^2^ A variety of oxidation products can be formed during this reaction. Hydroperoxides can decompose, producing alcohols, ketones, and organic acids. Additionally, the products generated can also polymerize forming gums that can affect biodiesel stability and performance.^6–8^

Since the reaction cannot be stopped once initiated, synthetic antioxidants are widely used to delay the reaction by inhibiting the formation of free radicals.^9,10^ However, there is growing interest in using plant-derived extracts with antioxidant properties as environmentally friendly alternatives. Several studies have been conducted employing different types of extracts such as oregano,^11^ black tea,^12^ araçá pulp,^13^ blackberry,^14^ senna,^15^ and green tea,^16^ showing their efficiency in delaying the biodiesel oxidation reaction.

Coffee leaves are particularly rich in polyphenolic compounds with potent antioxidant properties, making them a promising material for enhancing the oxidative stability of biodiesel.^17–19^ Compounds such as chlorogenic acids and flavonoids present in coffee leaves can act as free radical scavengers.^20,21^

In addition to antioxidant-based strategies, recent research has also emphasized the valorization of waste biomass for biodiesel production. Waste cooking oils, for example, have been successfully converted into high-quality biodiesel, especially when combined with partial hydrogenation to improve oxidative stability.^22^ Other promising approaches for enhancing biodiesel performance include the use of bifunctional heterogeneous catalysts, deep eutectic solvents (DES), and process integration techniques, such as the combination of transesterification and hydrogenation in a single step. These innovations not only improve the physicochemical properties of biodiesel but also promote greener and more efficient production methods.^23,24^

In this context, biodiesel degradation can be analyzed by using various techniques. The Rancimat method is standardized by EN 14112.^25^ It is used to determine the biodiesel oxidative stability by measuring the induction period considering the formation of volatile acid compounds in the sample but only provides a stability parameter in hours.^26^ Fourier Transform Infrared Spectroscopy (FTIR) can be used to detect changes in functional groups.^27^ Gas Chromatography coupled with Mass Spectrometry (GC-MS) is widely used for biodiesel characterization since it can provide the identification of volatile degradation products; however, it is time-consuming for sample preparation.^28^ In contrast, Nuclear Magnetic Resonance (NMR) spectroscopy allows a nondestructive, rapid, and direct analysis. It has been widely recognized for its effectiveness in identifying and quantifying lipid oxidation products.^29–34^ This technique is particularly useful in tracking shifts in the chemical environment of protons in fatty acid chains, providing molecular information about unsaturations as well as the formation of primary and secondary oxidation products.^35,36^

This study aimed to investigate the thermal-oxidative degradation of biodiesel, both with and without the addition of coffee leaf extract as a natural antioxidant, by applying proton nuclear magnetic resonance (^1^H NMR) spectroscopy. The approach involved monitoring the formation and evolution of oxidation products throughout the degradation process and establishing a correlation between the proportion of olefinic protons identified in the ^1^H NMR spectra and the iodine value (IV) determined by the Wijs method focusing on a rapid and nondestructive alternative for evaluating biodiesel stability.

## 2 Materials and Methods

### 2.1 Biodiesel Production

The biodiesel B100 used in the experiments was provided by the Fuel Research and Analysis Laboratory of the State University of Londrina, Brazil. The material was obtained by the triglyceride transesterification reaction using a mixture of 50% palm oil (S.S. Moratto Comércio de Insumos, São Paulo/SP, Lot SE-0518/25672) and 50% soybean oil (Coamo, Lot 423058). The reaction was conducted with absolute methanol (F.Maia, PA 99.8%) and potassium hydroxide (Sigma-Aldrich, 95%) as a catalyst. The molar ratio of alcohol to triglyceride was 6:1. The synthesis was performed under reflux at 60 °C with continuous stirring for 2 h. After phase separation, the biodiesel was washed first with an aqueous hydrochloric acid solution (1.5% w/w) and subsequently with deionized water, both heated to 80 °C, until a neutral pH was reached. The biodiesel was then dried to remove the residual moisture. The biodiesel production followed the methodology described by Branco et al.^37^

### 2.2 Physical and Chemical Characterization of Biodiesel

The biodiesel was characterized according to standardized methods. Density at 20 °C was measured following ASTM D4052,^38^ flash point by ASTM D93,^39^ and kinematic viscosity at 40 °C by ASTM D445.^40^ The ester content was determined by EN 14103.^41^ The content of mono-, di-, and triglycerides, as well as free and total glycerin, was analyzed following ASTM D6584.^42^ The acid number was determined by ASTM D664,^43^ water content by ASTM D6304,^44^ and cloud and pour points were measured according to ASTM D2500.^45^

### 2.3 Coffee Leaf Natural Extract Production and Phenol Content Determination

The alcoholic extract of arabica coffee leaves (*Coffea arabica*) (SisGen Registry ABA1234) was produced at the Fuel Research Laboratory in Londrina-PR, Brazil. The coffee leaves were collected at the State University of Londrina (−23.327877, −51.200190). The extract was prepared by mixing 10 g of the dried sample with 250 mL of absolute ethanol and storing the mixture in the dark for 48 h. After this period, the solution was filtered by using quantitative filter paper (UNIFIL). The filtrate was then concentrated on a heating plate at 60 °C to approximately 50 mL and transferred to 50 mL volumetric flasks, and the final volume was adjusted with absolute ethanol.

For each 100 g of biodiesel, 9.75 mL of coffee leaf extract was added. This volume was determined from preliminary tests to guarantee that the biodiesel would achieve an induction period equal to or greater than 8 h, which is the value established by the international standard EN 14214.^46^ Before using the extract in biodiesel samples, the alcohol was removed through evaporation using a heating plate at 60 °C.

In order to determine the total phenolic compounds in coffee leaf extract, it were used the Folin–Ciocalteu method and a UV–vis spectrometer (Thermo Scientific, Model: Evolution 60) at a wavelength of 760 nm. The phenol content was expressed in mg gallic acid equivalent (GAE) per gram of dry matter.^15^

### 2.4 Sample Thermal Oxidation

Each prepared sample underwent an accelerated heating test at 110 °C, using the Rancimat equipment (Metrohm brand; model 873), with an airflow rate of 10 dm^3^ h^–1^, following the methodology described in the international standard EN 14112.^25^ The induction periods were determined from the inflection point of the conductivity curve as a function of time, generated by the equipment’s software.

Before the ^1^H NMR analyses were conducted, the samples were degraded using the accelerated heating method. A total of eight samples of the B100, pure biodiesel, were placed in the Rancimat equipment at 110 °C (B100-0, B100-1, B100-2, B100-3, B100-4, B100-5, B100-6, B100-7). Samples were removed at eight different time intervals to follow the degradation process. The B100-0 sample was kept in the equipment until the achievement of the induction period (IP), that is, when the inflection point of the conductivity curve as a function of time was reached. The samples were then transferred to glass tubes, cooled, and stored under refrigeration for further NMR analysis. The sampling intervals for each sample were determined based on preliminary tests. The degradation process for pure biodiesel samples is shown in Figure 1.

**Figure 1:**
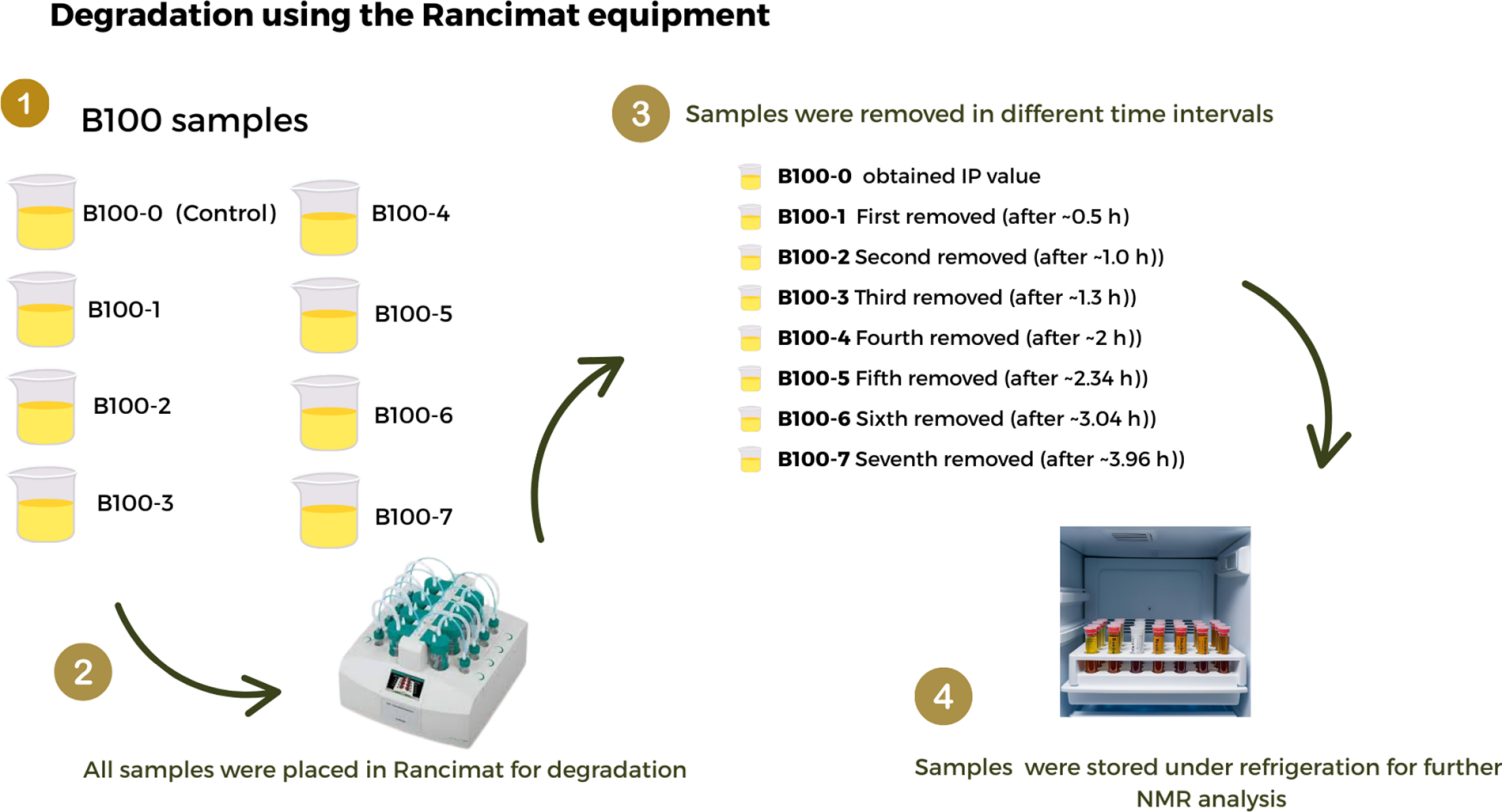
Flow diagram showing how pure biodiesel samples were separated, degraded, and stored before NMR analysis (created with Canva Pro. Elements used under Canva’s content license).

Similarly, a total of 11 samples of the B100E, biodiesel with coffee leaf extract, were placed in the Rancimat equipment at 110 °C (B100E-0, B100E-1, B100E-2, B100E-3, B100E-4, B100E-5, B100E-6, B100E-7, B100E-8, B100E-9, and B100E-10). Samples were removed at 11 different time intervals to follow the degradation process. The B100E-0sample was kept in the equipment until the achievement of the induction period (IP), that is, when the inflection point of the conductivity curve as a function of time was reached. The samples were then transferred to glass tubes, cooled, and stored under refrigeration for further NMR analysis. The sampling intervals for each sample were determined based on preliminary tests. The degradation process for biodiesel samples with coffee leaf extract is shown in Figure 2.

**Figure 2:**
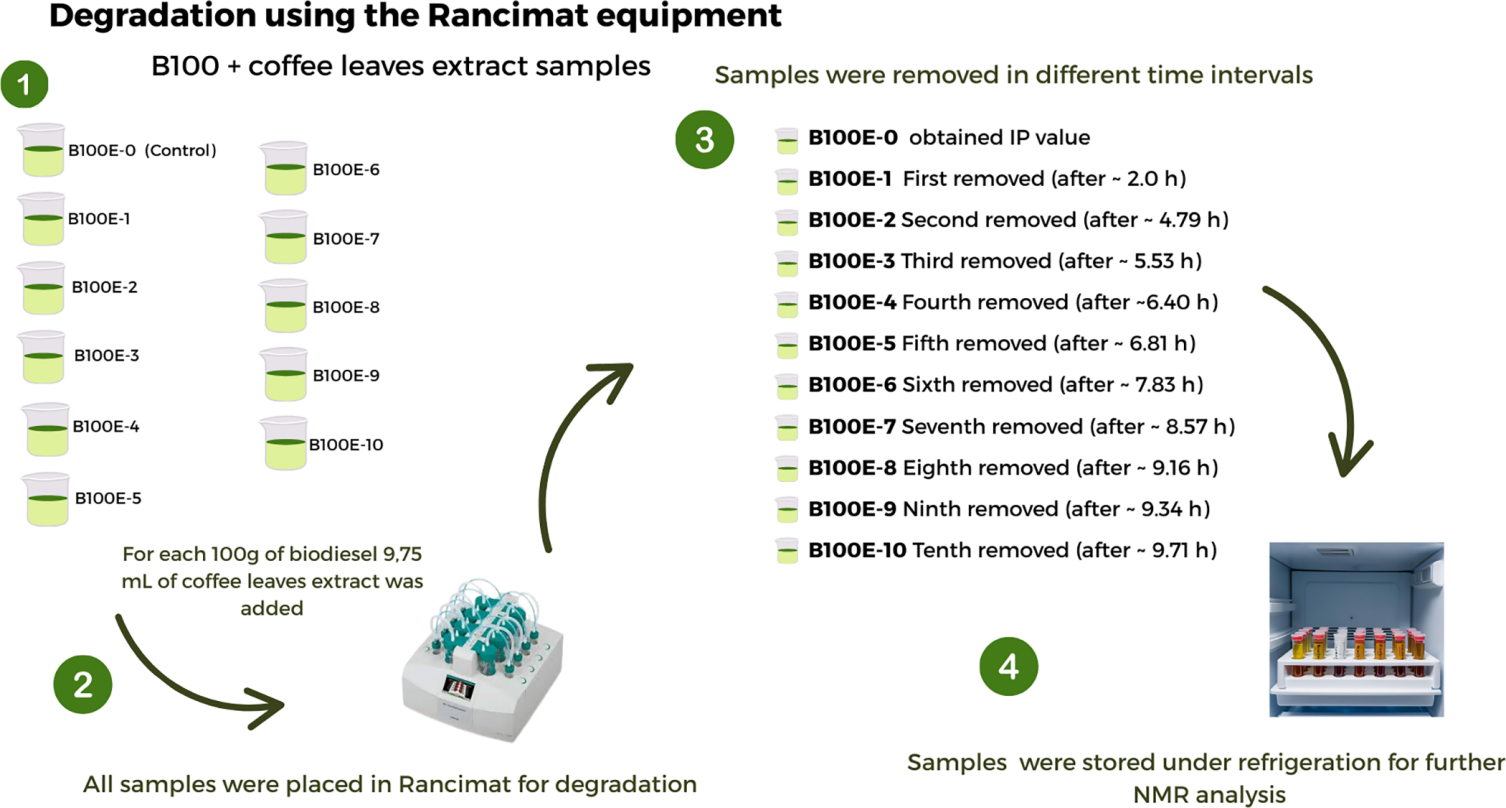
Flow diagram showing how biodiesel with coffee leaf extract samples were separated, degraded, and stored before NMR analysis (created with Canva Pro. Elements used under Canva’s content license).

### 2.5 Induction Period (IP)

The control samples were subjected to the accelerated heating method at 110 °C using a Rancimat apparatus (brand: Metrohm; model: 873), with an airflow rate of 10 dm^3^ h^–^^1^, according to EN 14122.^25^ The induction periods (IP) were determined by the inflection point of the electric conductivity versus time curve generated by the software.

The induction periods for the other samples could not be obtained, as they fully degraded within the equipment, preventing the measurement of a complete conductivity curve. Consequently, only the control samples provided the induction period values.

### 2.6 ^1^H NMR Spectroscopy

Spectral acquisition was conducted using a high-resolution NMR spectrometer operating at 9.4 T and 400 MHz (brand: Bruker). For sample preparation, 50 μL of biodiesel was dissolved in 600 μL of deuterated chloroform (CDCl_3_ 99.8% with 0.05% (v/v) tetramethylsilane—TMS, Sigma-Aldrich). The experimental parameters included single-pulse excitation, spectral width of 8012 Hz, 16 scans, a relaxation delay of 1 s, a 90° pulse width, and an acquisition time of 4.089 s. Chemical shifts were reported in ppm, with TMS serving as the internal standard.

All spectra underwent zero-order phase alignment and baseline correction within the spectral range from δ = −2.00 to 10.00 ppm, using TopSpin software (version 3.6.1). The integration of spectral signal regions corresponding to the CH_3_ acyl chains of saturated, oleate, and linoleate protons (0.83–0.93 ppm) (A), polyunsaturated fat (PUFA) protons (0.93–1.03 ppm) (B), allylic protons (1.8–2.2 ppm) (C), methylene groups α to the carbonyl protons (2.2–2.4 ppm) (D), bis-allylic protons (2.6–3.0 ppm) (E), and olefinic protons (5.0–5.7 ppm) (F) was performed using a Matlab routine (version 2015). The regions used in each spectrum are shown in Figure 3.

**Figure 3:**
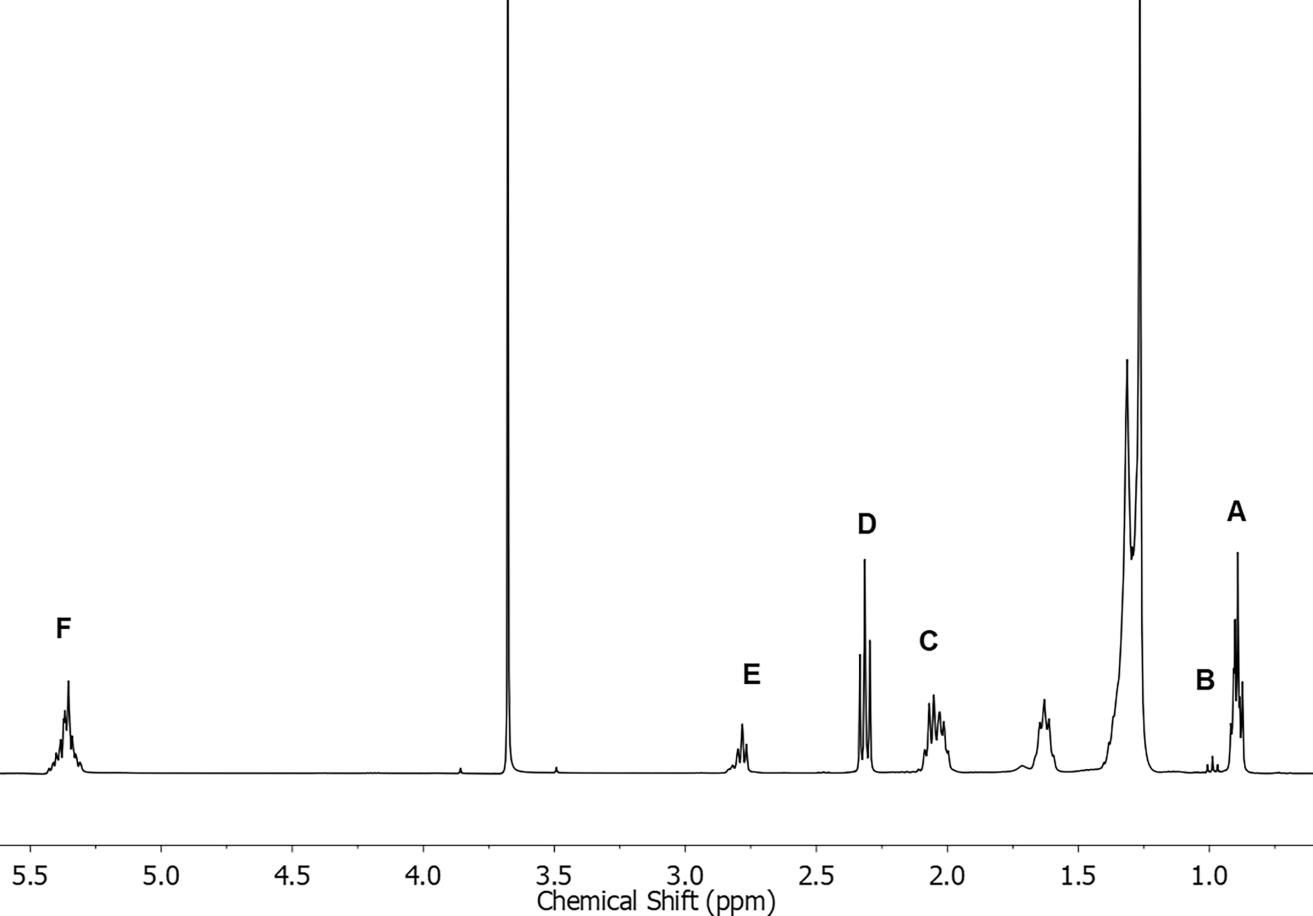
Biodiesel ^1^H NMR spectrum (400 MHz, CDCl, room temperature) highlighting the integration of spectral regions corresponding to CH_3_ acyl chains of saturated, oleate, and linoleate protons (0.83–0.93 ppm) (A); polyunsaturated fat (PUFA) protons (0.93–1.03 ppm) (B); allylic protons (1.8–2.2 ppm) (C); methylene groups α to the carbonyl (2.2–2.4 ppm) (D); bis-allylic protons (2.6–3.0 ppm) (E); and olefinic protons (5.0–5.7 ppm) (F).

The area of methylene groups α to the carbonyl (six protons), originally present in triglyceride molecules, was used to normalize the results. The integration intervals, previously described in the literature were adapted for this study.^29^

### 2.7 Iodine Value Determination

Iodine values were determined by the Wijs method.^47^ Approximately 0.25 g of each sample was weighed into a 500 mL Erlenmeyer flask with a stopper, followed by the addition of 10 mL of carbon tetrachloride. Then, 25 mL of Wijs solution was transferred to the flask containing the sample. The flask was capped and gently agitated with rotational movement to ensure thorough homogenization. The mixture was then left to rest in the dark at room temperature for 30 min. After this period, 10 mL of 15% potassium iodide solution and 100 mL of freshly boiled and cooled water were added. The solution was titrated with 0.1 M sodium thiosulfate until a faint yellow color appeared. Subsequently, 1 to 2 mL of 1% starch indicator solution was added, and titration continued until the blue color completely disappeared. A blank determination was prepared and processed in the same manner as for the samples to ensure accuracy.

## 3 Results and Discussion

### 3.1 Biodiesel Parameters

The biodiesel used presented a flash point value of 183.4 °C. The density obtained was 875.4 kg m^–3^ at 20 °C; the cloud point was 4 °C; and the acid number was 0.12 mg KOH g^–1^. The combined mono-, di-, and triglyceride contents together totaled 0.76% w/w, with a free glyceride content of 0.018% w/w. The water content obtained was 191.3 mg kg^–1^, and viscosity was 4.42 mm^2^ s^–1^. All parameters mentioned are within the specification parameters for B100 biodiesel according to current legislation in Resolution 920.^48^

### 3.2 Total Phenol Content of the Extract

The total phenol content of the alcoholic extract of coffee leaves was obtained using the Folin–Ciocalteu method and presented a value of 21.99 mg GAE g^–1^ dry mass (mg of gallic acid equivalents—GAE). Some researchers previously analyzed the influence of coffee leaf extract in the degradation of soybean biodiesel and obtained a phenolic content of 12.472 mg GAE g^–1^.^49^ It is important to note that factors such as extraction method, growing conditions, harvest time, and geographical location can directly impact in the raw material composition.^50^

When comparing the phenol content in coffee leaf extract with other plants, coffee leaves demonstrated competitive or superior antioxidant potential. The total phenolic content of various plant-based antioxidants has been investigated in several studies, the values of which are summarized in Table 1.

**Table 1:** Total Phenolic Content (TPC) of Coffee Leaf Extract Compared to Those of Other Plant Extracts Reported in the Literature

plant extract	TPC (mg GAE g^–1^ dry mass)	reference
coffee leaves	21.99	this study
bacuri peels	19.60	^13^
rosemary leaves	19.29	^13^
senna leaves	16.45	^15^
blackberry	4.62	^15^
hibiscus flower	4.06	^15^
araçá pulp	3.67	^13^

### 3.3 NMR Kinetics

NMR signals can be used to analyze biodiesel degradation, as they can provide detailed information on the functional group components of fatty acids. According to Guillén and Ruiz,^51^ the proportions of the degrees of unsaturation of different acyl groups can be determined from NMR, since the area of each signal in the spectrum corresponds to the number of hydrogens present in each sample. Thus, the proportions (in percentage) of the acyl groups linolenate (Ln), linoleate (L), oleate (O), and saturated (S) from all samples were calculated based on the equations presented by Guillén and Ruiz^51^ and are shown in Figures 4 and 5.

**Figure 4:**
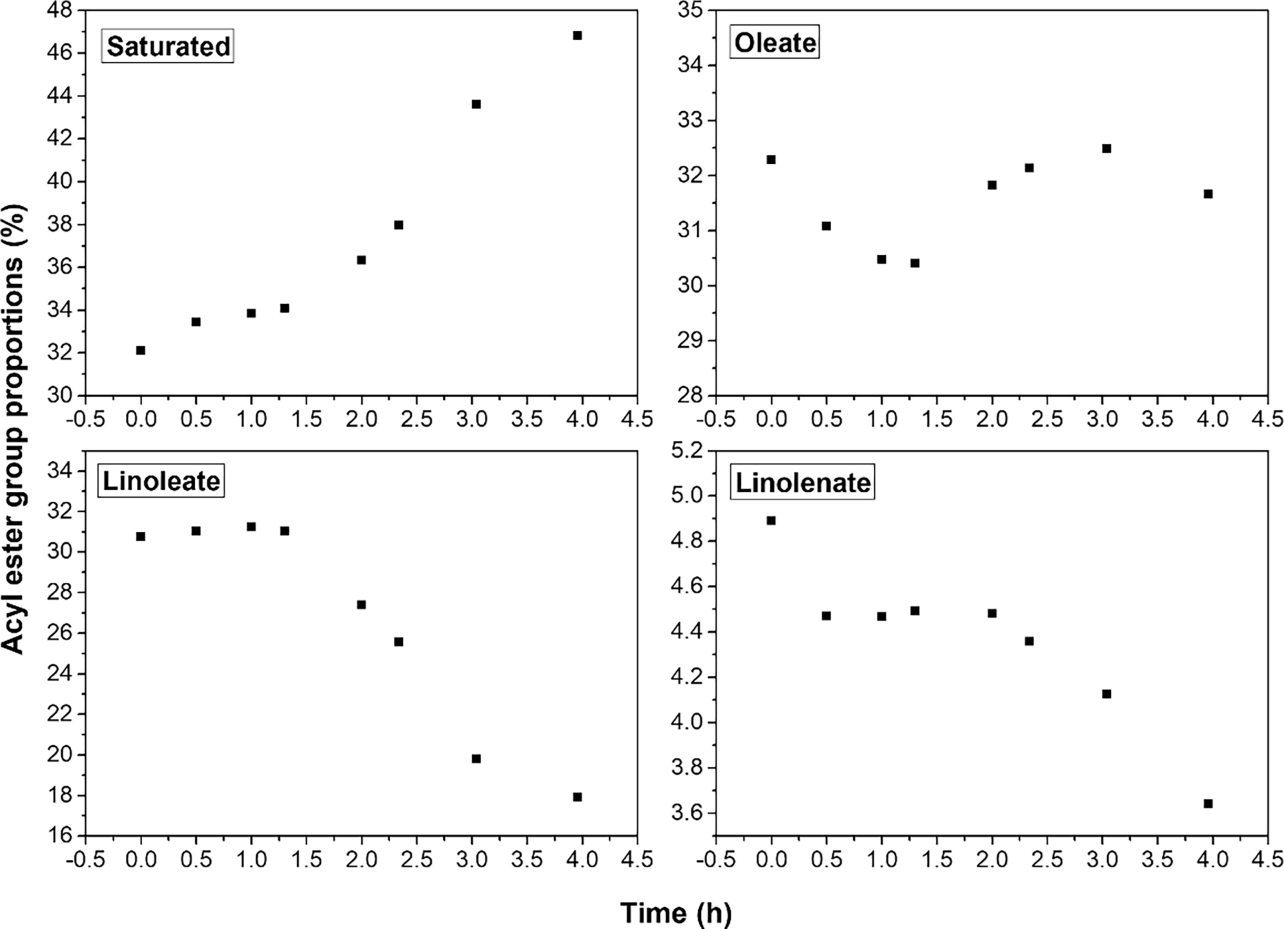
Methyl ester percentages of the acyl groups Saturated (S), Oleate (O), Linoleate (L), and Linolenate (Ln) of the pure biodiesel samples, degraded at 100 °C, with an induction period of 2.19 h.

**Figure 5:**
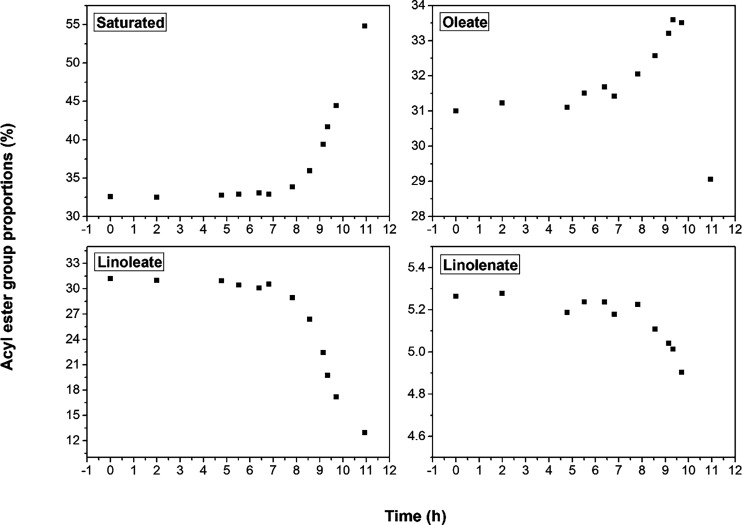
Methyl ester percentages of the acyl groups Saturated (S), Oleate (O), Linoleate (L), and Linolenate (Ln) of the biodiesel samples with coffee leaf extract, degraded at 100 °C, with an induction period of 8.9 h.

Based on the induction period data obtained using the Rancimat equipment, it can be observed that the extract of coffee leaves, with antioxidant properties, enhances the oxidative stability of biodiesel, increasing the induction period by 6.71 h.

Table 2 displays the percentage composition of acyl groups in the B100 biodiesel sample. During the degradation process, the percentages of linoleate and linolenate groups decreased by about 1.25 and 12.85%, respectively; the percentage of the oleate group remained approximately constant, while the percentage of the saturated compounds increased by approximately 14.7%. These results suggest that biodiesel degradation occurred as the number of saturated compounds increased throughout the degradative process.

**Table 2:** Methyl Esters Percentages of the Acyl Groups Saturated (S), Oleate (O), Linoleate (L), and Linolenate (Ln) of the Pure Biodiesel Samples Degraded at 100 °C

samples	S (%)	O (%)	L (%)	Ln (%)
B100-0^a^	32.08	32.28	30.74	4.89
B100-1	33.44	31.09	31.00	4.47
B100-2	33.84	30.48	31.21	4.47
B100-3	34.08	30.40	31.02	4.49
B100-4	36.32	31.82	27.38	4.48
B100-5	37.94	32.13	25.57	4.36
B100-6	43.60	32.49	19.78	4.13
B100-7	46.81	31.66	17.89	3.64

a Induction period = 2.19 h.

The degradative process of the biodiesel sample in the presence of coffee leaf extract with antioxidant properties can be observed in Table 3. This sample exhibits behavior similar to that of the pure B100 biodiesel sample but with a longer degradative process. Regarding the percentages of linoleate and linolenate groups, there are decreases of 48.56 and 0.34%, respectively. The oleate group initially experienced a decline in its percentage during the early stages of degradation but showed a final increase of 2.73%. This occurs because, throughout the degradation process, portions of the linoleate and linolenate proportions convert into oleate compounds.^33^ Finally, the proportion of saturated compounds increases by 11.81%, indicating that the oxidative degradation of the sample occurred.

**Table 3:** Methyl Ester Percentages of the Acyl Groups Saturated (S), Oleate (O), Linoleate (L), and Linolenate (Ln) of the Biodiesel Samples with the Coffee Leaf Extract Degraded at 100 °C

samples	S (%)	O (%)	L (%)	Ln (%)
B100E-0^a^	32.55	31.00	31.18	5.26
B100E-1	32.51	31.23	30.99	5.28
B100E-2	32.78	31.10	30.93	5.19
B100E-3	32.85	31.50	30.41	5.24
B100E-4	33.02	31.68	30.06	5.24
B100E-5	32.87	31.42	30.53	5.18
B100E-6	33.81	32.05	28.92	5.22
B100E-7	35.95	32.57	26.38	5.11
B100E-8	39.35	33.20	22.41	5.04
B100E-9	41.69	33.59	19.71	5.01
B100E-10	44.41	33.51	17.18	4.90

a Induction period = 8.9 h.

A comparison between the two samples reveals that both underwent degradation, primarily indicated by the increase in the proportions of the saturated compounds. However, the degradation times differed between them. The sample containing the natural antioxidant from coffee leaves exhibited a longer degradation period, suggesting the protective effect of the antioxidant in biodiesel. Molecules with antioxidant properties are expected to inhibit the oxidative process and maintain the composition of fatty acid constant.^34^ The data indicate that up to the sample B100E-6, the proportions remained approximately constant, and beyond this point, a noticeable decline or increase was observed, indicating the antioxidant’s role in delaying the oxidation process. Furthermore, the B100 sample showed a 14.7% increase in the number of saturated compounds, whereas the B100E sample exhibited an increase of 11.81%, further confirming the effectiveness of the extract in reducing the formation of saturated compounds during the oxidative process.

The protective effect observed in the B100E samples can be attributed to molecular-level interactions between the antioxidant compounds in the coffee leaf extract and reactive oxidative species. Phenolic compounds, such as chlorogenic acids and flavonoids, may act by donating hydrogen atoms from their hydroxyl groups to lipid radicals, forming resonance-stabilized phenoxyl radicals that interrupt the propagation of the oxidative chain reaction. Additionally, these compounds may chelate trace metal ions present in biodiesel, reducing their catalytic role in hydroperoxide decomposition. The antioxidant activity observed is consistent with these well-established mechanisms of radical scavenging and metal chelation reported for plant-derived phenolics.^50,52^

### 3.4 Degradation Product Identification

Oxidation products can be identified using NMR spectroscopy, as some authors have already mentioned.^29,30,34^ Thus, the biodiesel samples were analyzed, and the identification of oxidation products was performed. The identified oxidation products are shown in Table 4 and Figures 6 and 7.

**Table 4:** Chemical Shifts of Oxidation Products Detected in the Biodiesel Samples during the Degradation Process

chemical shift (ppm)	attribution	functional group	reference
2.58–2.53	epoxides	–C**H**OHC–	^36^
		9,10–12,13-diepoxyoctadecanoate	
2.99–2.88^a^	epoxides	–C**H**O**H**C–	
		9,10-epoxy-octadecanoate	
		9,10-epoxy-12-octadecenoate(leukotoxin); 12,13-epoxy-9-octadecenoate(isoleukotoxin)	
3.16–3.10	epoxides	–CHO**H**C–CH_2_–C**H**OHC–	^53^
		9,10–12,13-diepoxy-octadecanoate	
4.40–4.25	hydroperoxides	>C**H**–OOH (methine proton of hydroperoxide group)	^54^
5.90–5.72	hydroperoxide	–C**H**=C**H**–C**H**=C**H** (*cis, trans* conjugated diene groups)	^33,55^
		(*Z*,*E*)-conjugated double bonds associated with hydroxides (OH)	
		double bond associated with hydroperoxides (OOH)	
6.11–5.99	hydroperoxides	–C**H**=C**H**–C**H**=C**H** (*cis, trans* conjugated diene groups)	^4,54^
6.34–6.22	hydroperoxide derivatives	–C**H**=C**H**–C**H**=C**H**–	^4,55^
		(*E*,*E*)-conjugated double bonds associated with hydroperoxides (OOH)	
6.65–6.50	hydroperoxide	–C**H**=C**H**–C**H**=C**H** (*cis, trans* conjugated diene groups)	^4,54^
6.90–6.80	phenolic compounds	–Ph–**H** (phenolic ring)	^54^
8.40–8.00	hydroperoxide	–OO**H** (hydroperoxide group)	^54^
9.80–9.45	aldehydic group	–C**H**O aldehydic group	^4,55^
		(*E*)-2-alkenals	
		(*E*,*E*)-2,4-alkadienals	
		4,5-epoxy-2-alkenals	
		4-hydroxy-(*E*)-2-alkenals	
		4-hydroperoxy-(*E*)-2-alkenals	
		(*Z*,*E*)-2,4-alkadienal	
		*n*-alkanals	
		4-oxo-alkanals	
		*n*-alkanals of low molecular weight (ethanal and propanal)	

a It is important to notice that this signal presents an overlap.

**Figure 6:**
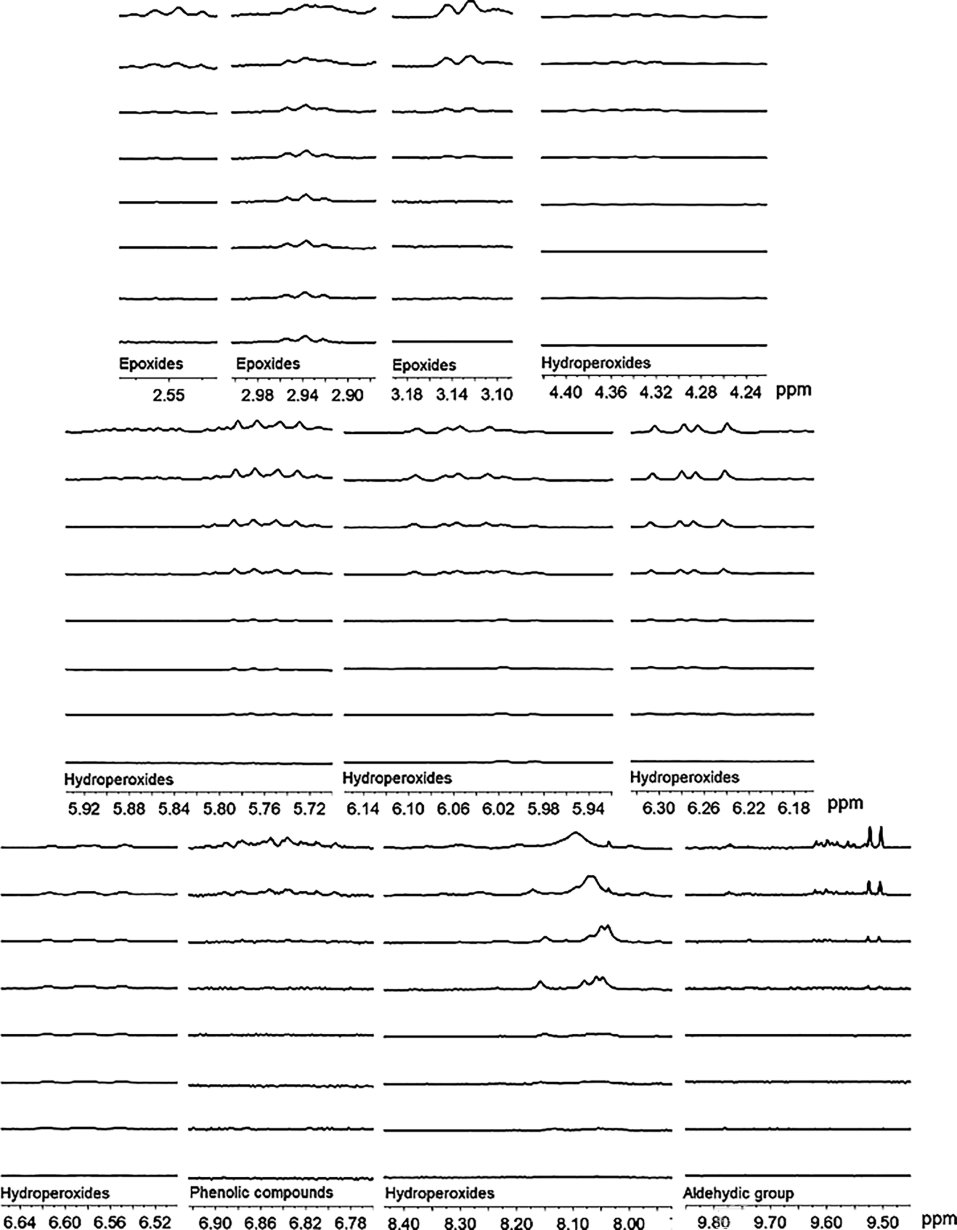
Oxidation products were detected during the degradation of pure biodiesel samples. The bottom line represents the B100-0 sample, while the subsequent lines correspond to B100-1, B100-2, and so on, following the progression of degradation.

**Figure 7:**
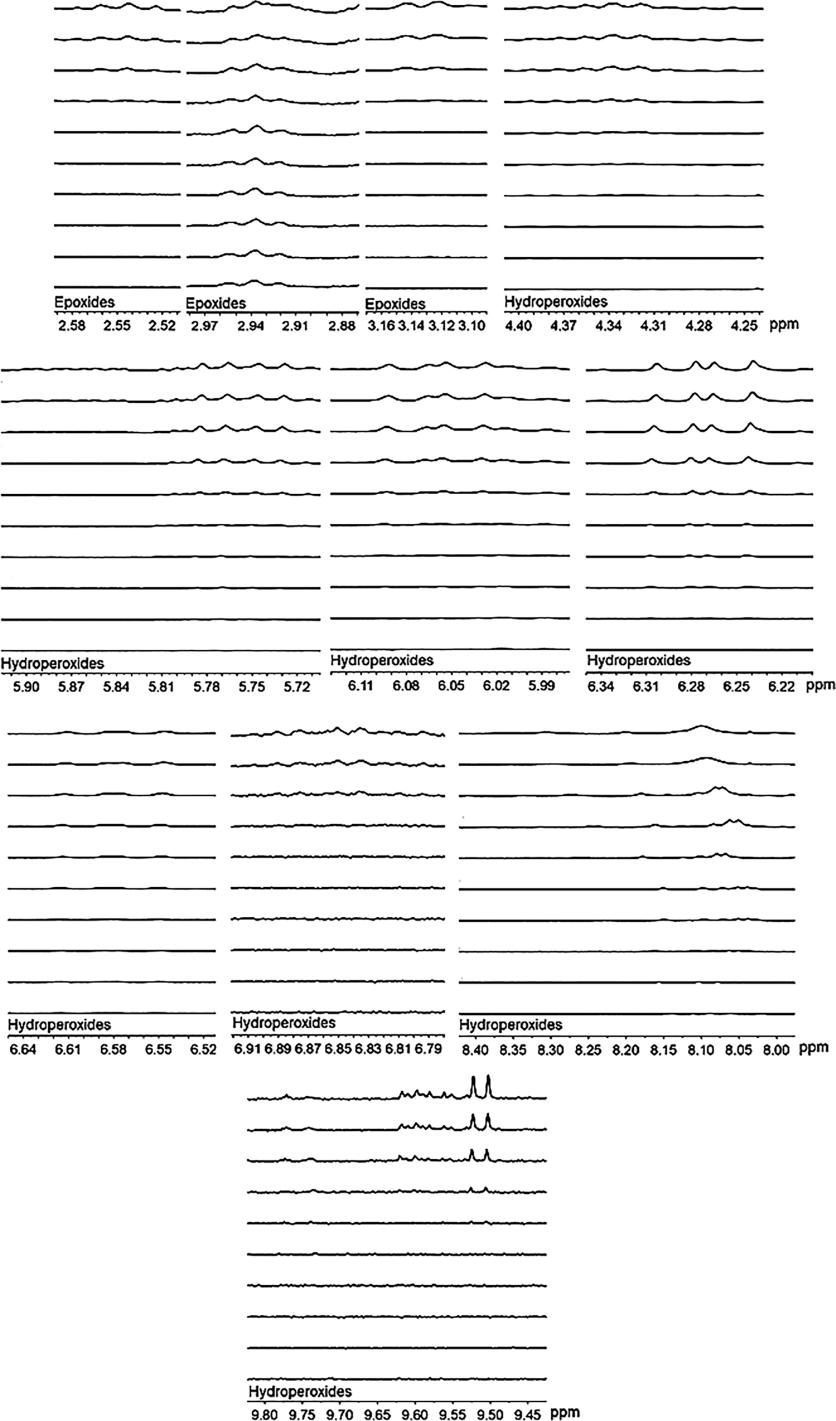
Oxidation products detected during the degradation of biodiesel samples with coffee leaf extract. The bottom line represents the B100E-0 sample, while the subsequent lines correspond to B100E-1, B100E-2, and so on, following the progression of degradation.

Analyzing oxidation products formed in the biodiesel samples (Table 4), both primary and secondary oxidation products were observed, including epoxides, hydroperoxides, and aldehydes. These products represent the typical oxidative breakdown of fatty acid chains in biodiesel. Primary oxidation products, such as hydroperoxides, are generated in the initial stages of degradation, while secondary products, such as aldehydes and epoxides, result from further decomposition of these compounds.^56,57^

The presence of epoxides reflects the attack on double bonds in unsaturated fatty acids, leading to cyclization reactions.^53^ Hydroperoxides indicate the initial steps of oxidation, where oxygen is incorporated into the lipid structure.^55^ Aldehydes and alkenals, observed at higher ppm ranges, are secondary oxidation products, often responsible for significant degradation of biodiesel quality, contributing to poor stability and performance.^58^

From Figures 6 and 7, it is evident that the oxidation products formed during the degradation of B100 (Figure 6) and B100E (Figure 7) include similar compounds. However, a notable difference is observed in the B100E sample, where the appearance of these products occurs later in the NMR signals, indicating a slower progression of the degradation process. This delay suggests that the coffee leaf extract present in the B100E sample effectively acts as an antioxidant, delaying the oxidation reaction by stabilizing the fuel and reducing the formation of free radicals that initiate degradation. Consequently, the antioxidant properties provided in the extract contribute to enhanced oxidative stability, as evidenced by the later detection of oxidation products compared to the pure biodiesel sample.

### 3.5 Iodine Value Determination

The iodine value (IV) is one of many parameters that can be used to analyze biodiesel degradation, since it represents the unsaturated methyl esters present in the material, directly impacting its oxidative stability. The IV is defined as the mass of iodine absorbed in 100g of biodiesel sample. The standard EN 14214 specifications limit the IV in biodiesel to 120.^46^ Additionally, the IV indicates the total unsaturation content in biodiesel.^59^

Over the past decades, some researchers have proposed some methods to evaluate the iodine value of oil samples using the ^1^H NMR spectra by analyzing the signal of the olefinic protons. All of them presented satisfactory results.^51,60,61^

In this study, the iodine values for the biodiesel samples were obtained through the Wijs method compared to the proportion of olefinic hydrogen atoms in each sample during the degradation process.^62^ The correlation between the IV (Wijs method) and the olefinic proportions for both samples, pure biodiesel and biodiesel with coffee leaf extract, is shown in Figures 8 and 9.

**Figure 8:**
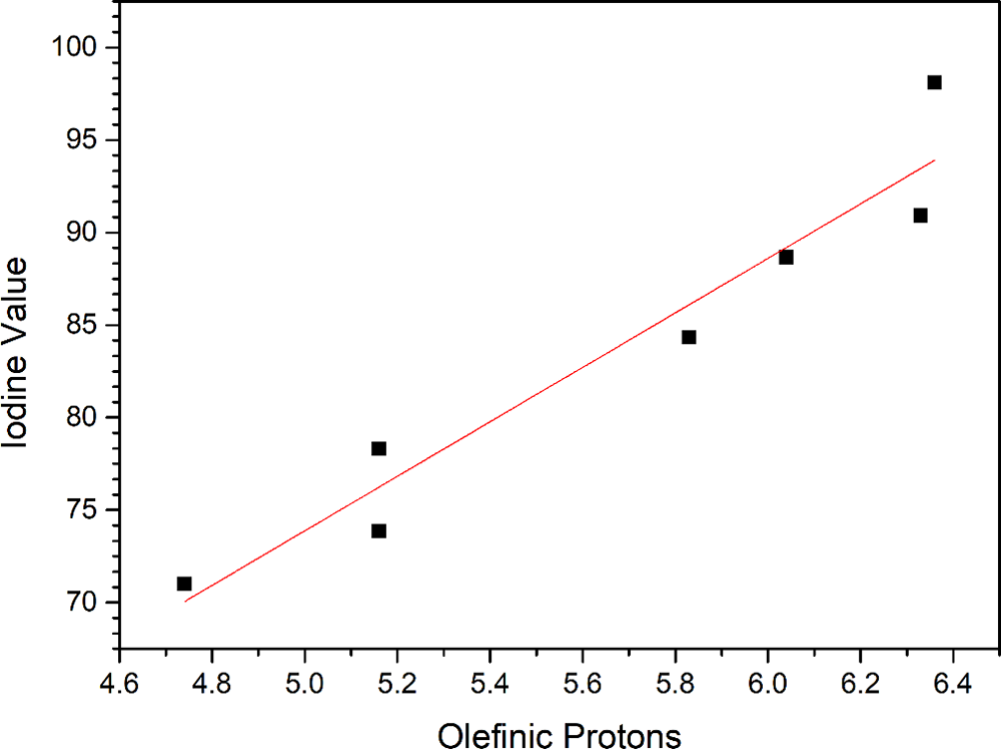
Correlation between the percentage of olefinic hydrogen atoms from the ^1^H NMR spectra and the iodine value determined by the Wijs method for the pure biodiesel samples.

**Figure 9:**
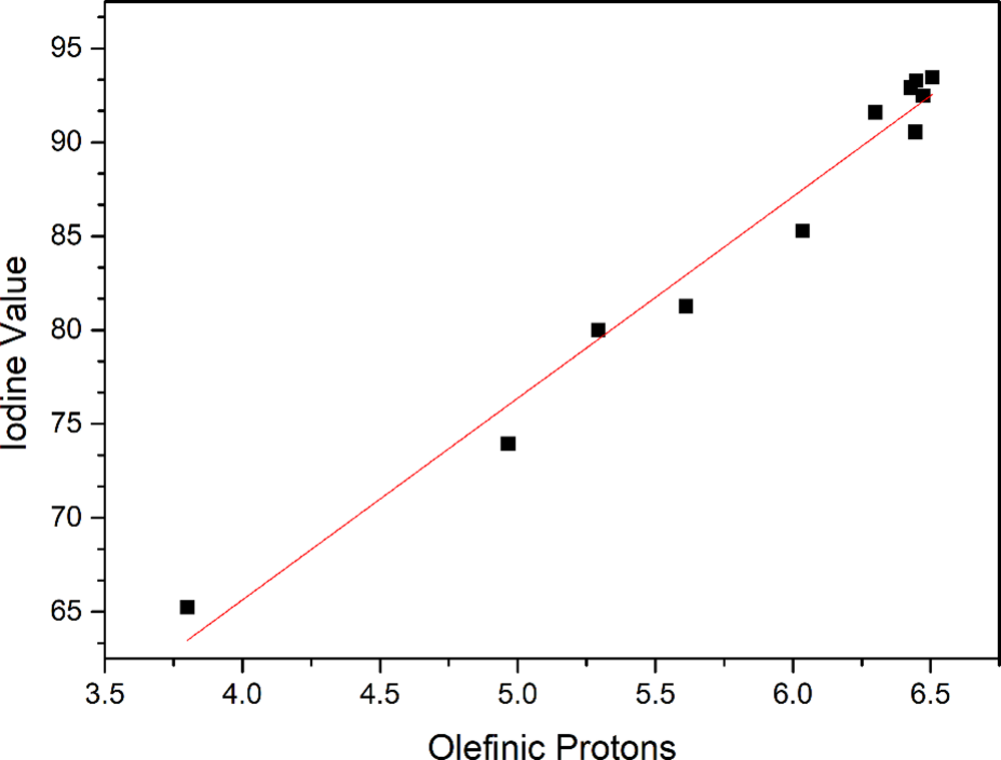
Correlation between the percentage of olefinic hydrogen atoms from the ^1^H NMR spectra and the iodine value determined by the Wijs method of the biodiesel samples with coffee leaf extract.

Considering Figures 8 and 9, both samples present a linear correlation between the percentage of olefinic hydrogen atoms and the iodine value. These correlations are shown by the following linear equations: **IV**_B100_ = 0.22 + 14.73**O** (*R* = 0.97) for pure biodiesel and **IV**_B100FC_ = 22.66 + 10.74**O** (*R* = 0.99) for biodiesel with the coffee leaf extract.

According to Guillén and Ruiz,^51^ the proportion of olefinic hydrogens and the IV provide the same information. Therefore, ^1^H NMR spectra can be used as a direct determination of the unsaturation degree of oils and fats and also biodiesel. This approach is particularly advantageous since it allows a rapid and straightforward determination of IV, unlike conventional methods, making it a practical alternative for analyzing unsaturation levels.

## 4 Conclusions

This study investigated the thermal-oxidative degradation of biodiesel, both with and without the addition of coffee leaf extract, using ^1^H NMR spectroscopy to monitor molecular changes during the oxidation process.

The kinetic analysis of the degradation revealed that linolenate and linoleate compounds exhibited the fastest oxidation rates with a significant reduction in their proportion as degradation progressed. Oleate compounds displayed a more stable behavior, with some being converted from linoleate and linolenate structures during oxidation. This transformation further supports the observed increase in the number of saturated compounds over time, confirming the expected oxidation process.

The results also demonstrated that oxidation products, such as epoxides, hydroperoxides, and aldehydes, were formed during the degradation of both biodiesel samples. The presence of coffee leaf extract effectively delayed the oxidation, as indicated by the later appearance of oxidation products in the B100E sample compared to pure biodiesel B100. The increase in saturated compounds was also lower in the B100E sample, confirming the antioxidant effect of the extract.

The ^1^H NMR analysis proved to be a valuable tool for monitoring biodiesel degradation, as it provided quantitative insights into the oxidation process. Additionally, the correlation between the iodine value (IV) and olefinic hydrogen content confirmed that NMR spectroscopy can serve as a rapid and efficient alternative method for assessing the unsaturation degree of biodiesel.

The findings suggest that coffee leaf extract is a promising natural antioxidant, enhancing the oxidative stability of biodiesel and offering a sustainable alternative to synthetic additives.
